# Growth differentiation factor 15 in a community-based sample: age-dependent reference limits and prognostic impact

**DOI:** 10.1080/03009734.2018.1460427

**Published:** 2018-05-01

**Authors:** Steven Doerstling, Pär Hedberg, John Öhrvik, Jerzy Leppert, Egil Henriksen

**Affiliations:** a Center for Clinical Research, Uppsala University, Västmanland County Hospital, Västerås, Sweden; b Department of Clinical Physiology, Västmanland County Hospital, Västerås, Sweden

**Keywords:** All-cause mortality, GDF-15, reference values, survival analysis, protein biomarkers

## Abstract

**Background:**

Despite the growing body of evidence on growth differentiation factor 15 (GDF-15) reference values for patients with existing cardiovascular disease, limited investigation has been dedicated to characterizing the distribution and prognostic impact of GDF-15 in predominantly healthy populations. Furthermore, current cutoff values for GDF-15 fail to account for the well-documented age-dependence of circulating GDF-15.

**Methods:**

From 810 community-dwelling older adults, we selected a group of apparently healthy participants (*n* = 268). From this sample, circulating GDF-15 was modeled using the generalized additive models for location scale and shape (GAMLSS) to develop age-dependent centile values. Unadjusted and adjusted Cox proportional hazards models were used to assess the association between the derived GDF-15 reference values (expressed as centiles) and all-cause mortality.

**Results:**

Smoothed centile curves showed increasing GDF-15 with age in the apparently healthy participants. An approximately three-fold difference was observed between the 95th and 5th GDF-15 centiles across ages. In a median 8.0 years of follow-up, 97 all-cause deaths were observed in 806 participants with eligible values. In unadjusted Cox regression analyses, the hazard ratio (95% CI) for all-cause mortality per 25-unit increase in GDF-15 centile was 1.80 (1.48–2.20) and dichotomized at the 95th centile, ≥95th versus <95th, was 3.04 (1.99–4.65). Age-dependent GDF-15 centiles remained a significant predictor of all-cause mortality in all subsequent adjusted models.

**Conclusions:**

Age-dependent GDF-15 centile values developed from a population of apparently healthy older adults are independently predictive of all-cause mortality. Therefore, GDF-15 reference values could be a useful tool for risk-stratification in a clinical setting.

**ClinicalTrials.gov Identifier:**

NCT01452178.

## Introduction

Recent scholarship has called attention to the importance of growth differentiation factor 15 (GDF-15) as a protein biomarker with valuable prognostic utility for predicting adverse outcomes in patients with cardiovascular disease (CVD). GDF-15 is a member of the transforming growth factor-beta superfamily of cytokines and is expressed in low quantities across most cell types under physiological conditions (1–3). Expression of GDF-15 is strongly induced by pro-inflammatory cytokines (1) and cellular insults such as ischemia (4), oxidative stress (5,6), and biomechanical stress to the myocardium (7). Accordingly, circulating levels of GDF-15 are characteristically elevated in patients with neoplastic disease, heart failure, and acute coronary syndrome (2,8–10). The precise mechanisms by which GDF-15 exerts its bioactivity in these conditions remains uncertain. However, evidence suggests that GDF-15 functions as an autocrine anti-inflammatory and cellular repair factor (1,4,11,12), secreted in quantities commensurate to acute and chronic tissue injury (3,13).

In both the general population and in the context of existing CVD, circulating GDF-15 is associated with a variety of risk factors and biomarkers for cardiovascular outcomes including age, blood pressure, diabetes mellitus, and N-terminal pro-brain natriuretic peptide (NT-proBNP) (2,10,14–16). Despite the growing body of evidence on GDF-15 reference values for patients with existing CVD, limited investigation has been dedicated to characterizing the distribution and prognostic impact of GDF-15 in predominantly healthy populations. Considering the mounting interest in prognostic biomarkers to support risk-stratification and clinical decision-making, the aim of the present study was two-fold: to determine reference values for the distribution of GDF-15 in a population of apparently healthy older adults and evaluate the degree to which the reference values predict all-cause mortality.

## Methods

### Study population

The participants were recruited from the control group of the Västmanland Myocardial Infarction Study (VaMIS; ClinicalTrials.gov Identifier: NCT01452178). A comprehensive description of the control group has been reported elsewhere (17). Briefly, patients hospitalized for acute myocardial infarction at Västmanland County Hospital, Västerås, Sweden from November 2005 to May 2011 were consecutively included in the VaMIS study. Control subjects were continuously recruited from the general population during the inclusion of the VaMIS patients and matched 1:1 by closest date of birth, same sex, and same municipality to each patient.

### Data collection

All subjects underwent clinical examination, echocardiographic evaluation, and blood sampling.

At the baseline examination, demographics and medical history were collected. Patient-reported diagnoses of myocardial infarction (MI), transient ischemic attack (TIA)/stroke, and diabetes mellitus were confirmed from medical records. History of heart failure, cancer, hypertension, and medication use were self-reported. By the use of echocardiography, left ventricular systolic dysfunction was defined as having a left ventricular ejection fraction (LVEF) of <45% and significant valvular disease as having moderate to severe stenosis or regurgitation in the aortic or mitral valves. Peripheral artery disease was determined present if the participant had an ankle–brachial index (ABI) value <0.91 or >1.39 (18). Non-sinus rhythm was determined by electrocardiography. Other major electrocardiographic (ECG) changes were based on the Minnesota code (MC) and determined present if any of the following conditions were met: Q wave (MC 1.1–1.2), ST segment depression (MC 4.1–4.2), T wave change (MC 5.1–5.2), A-V conduction defect (MC 1.1–1.2), ventricular conduction defect (MC 1.1–1.2), or left ventricular hypertrophy (S wave amplitude in lead V1 + R wave amplitude in V5 or V6 > 3.5 mV combined with MC 4.1–4.2 or 5.1–5.2 in lead V5 or V6). Blood pressure was measured manually and is reported as the mean of two measurements on the left arm after five minutes of rest with the subject in a seated position. Information on smoking status and leisure-time physical activity was self-reported.

### Blood sampling procedure

Blood samples were taken in 5 mL lithium heparin-coated vacuum tubes and centrifuged at 2000*g* for 10 min (Becton Dickinson and Co.) or 2200*g* for 10 min (Vacuette, Greiner Bio-One). Plasma was reallocated to 5 mL plastic tubes and frozen at −70° Celsius within two hours. Plasma samples remained stored at −70° Celsius. Before analysis, the samples were thawed at room temperature, mixed and centrifuged at 3470*g* at 4° Celsius for 15 min, and robotically aliquoted into a microtiter plate by the Tecan Freedom Evolyzer system.

### Group selection

From the control group of the VaMIS study (*n* = 855), we excluded individuals with missing values for circulating GDF-15 (*n* = 39), missing values for group selection (*n* = 5), and extreme measurements of GDF-15 reflected by standard measurements and secondly by the Olink Proximity Extension Assay chip (Olink Bioscience) with otherwise standard demographics and medical history (*n* = 1). To characterize the distribution of GDF-15 in older adults of apparently good health, subjects with a history of MI (*n* = 32), TIA/stroke (*n* = 47), heart failure (*n* = 21), hypertension (*n* = 375), LVEF <45% (*n* = 20), significant valvular disease (*n* = 11), peripheral artery disease (*n* = 70), angina pectoris (*n* = 56), non-sinus rhythm (*n* = 37), major ECG changes (*n* = 134), cancer (*n* = 77), diabetes (*n* = 75), body mass index >35 (*n* = 21), and those taking regular cardiovascular medication (*n* = 399) were designated to the ‘unhealthy’ group (*n* = 542), and the remaining subjects were designated to the ‘apparently healthy’ group (*n* = 268), resulting in a total of 810 participants included in the present study. All participants provided written informed consent. The Ethics Committee of Uppsala, Sweden approved the study (Protocol number: 2005:169).

### Biochemistry

Plasma concentrations of GDF-15 were measured using a commercially available sandwich immunoassay using monoclonal antibodies and biotin-streptavidin separation (Elecys GDF-15 and Cobas e411; Roche Diagnostics, Germany). LDL cholesterol was estimated using Friedewald’s formula.

### End-point and follow-up

The primary end-point was all-cause mortality. All-cause mortality was selected as the end-point to account for the frequent uncertainty in attributing the cause of death in older adults as well as to extend previous lines of investigation between GDF-15 and all-cause mortality. Study participants were followed until 9 March 2017, with a median (1st to 3rd quartile) follow-up of 8.0 (6.8–9.4) years.

### Statistical analysis

Data are presented as median (interquartile range) for continuous variables and number (percentage) for categorical variables, as indicated. Differences in baseline characteristics and GDF-15 between healthy and unhealthy participants were tested using Mann–Whitney *U* and chi-square tests. Clinical and biochemical quantities stratified by GDF-15 quartiles were compared using Kruskal–Wallis and chi-square tests. Spearman rank correlation was used to evaluate the relationship between GDF-15 and continuous clinical and biochemical variables that reached statistical significance across GDF-15 quartiles. Reference values presented as centile curves were generated to describe GDF-15 values as a function of age using the generalized additive models for location scale and shape (GAMLSS). This relationship was modeled using the lognormal distribution. Ten-fold cross-validation was used to validate the GAMLSS model, with −2 × log(likelihood) of the fitted GAMLSS model calculated as the performance measure.

Cox proportional hazards models were used to assess the association between GDF-15 reference values and all-cause mortality. GDF-15 centile values corresponding to each participant’s age and GDF-15 values were calculated based on the centile curves; these values entered adjusted and unadjusted models as a continuous variable. Adjusted model 1 was adjusted for age and sex. For a subsequent adjusted model, best subset selection using a golden section search algorithm (BeSS package version 1.0.3) was used to determine the optimal subset of parameters among the following variables: body mass index, smoking status, systolic blood pressure, NT-proBNP, and cystatin C. The model that yielded the smallest Akaike information criterion (AIC) included the following explanatory variables: smoking status, NT-proBNP, and cystatin C. In Adjusted model 2, these covariates were included with GDF-15, age, and sex. Crude and adjusted Cox regression models were also calculated using GDF-15 centiles dichotomized at the 95th centile. A separate best subset selection with the dichotomized GDF-15 centile variable yielded an identical set of covariates. Age was included in adjusted models using a restricted cubic spline with three knots. NT-proBNP was log-transformed (logNT-proBNP) in adjusted models. The proportional hazard assumption was verified for all explanatory variables by inspection of scaled Schoenfeld residual plots and for the entire model by a test of independence between the scaled Schoenfeld residuals and time. Inspection of Martingale residual plots was used to verify the linearity assumption. Additionally, the Kaplan–Meier estimator was used to estimate the survival function of all-cause mortality by GDF-15 centile dichotomized at the 95th centile; differences in survival curves were compared using the log-rank test.

All statistical analyses were performed using R 3.4.3 (R Foundation for Statistical Computing, 2017, Vienna, Austria; http://www.r-project.org). Two-sided *P* values <0.05 were considered to be statistically significant.

## Results

### Baseline characteristics

Apparently healthy participants were younger, had a lower proportion of overweight (BMI 25–29.9) and obese (BMI ≥ 30) individuals, included fewer current or previous smokers, and engaged in more strenuous leisure-time physical activity compared with unhealthy participants (Table 1). Significant differences in systolic blood pressure and blood lipid profiles were also observed between the apparently healthy and unhealthy groups; healthy participants had higher total and LDL cholesterol compared with unhealthy participants. No significant differences in sex or diastolic blood pressure were observed between the apparently healthy and unhealthy groups. The distribution of GDF-15 was significantly different between apparently healthy and unhealthy participants (*P* < 0.0001; Supplementary Figure 1).

**Table 1.** Baseline characteristics of study participants.

Variable^a^	All controls (*n* = 810)	Apparently healthy (*n* = 268)	Unhealthy (*n* = 542)	*P* value^b^
Age (years)	68 (14)	63.5 (14)	70 (13)	<0.0001
Sex				0.5947
Female	238 (29.4)	75 (28)	163 (30.1)	
Male	572 (70.6)	193 (72)	379 (69.9)	
Body mass index (kg/m^2^)				<0.0001
<25	293 (36.2)	127 (47.4)	166 (30.6)	
25–29.9	386 (47.7)	113 (42.2)	273 (50.4)	
≥30	131 (16.2)	28 (10.4)	103 (19)	
Systolic blood pressure (mmHg)	145 (28)	140 (24.3)	147.8 (26.5)	<0.0001
Diastolic blood pressure (mmHg)	80 (13.8)	80 (11)	80 (16)	0.1108
Smoking status				0.0303
Never smoked	348 (43)	130 (48.5)	218 (40.2)	
Current or previous smoker	462 (57)	138 (51.5)	324 (59.8)	
Physical activity, leisure-time				<0.0001
Low to mild	514 (63.5)	138 (51.5)	376 (69.4)	
Moderate to strenuous	296 (36.5)	130 (48.5)	166 (30.6)	
Biochemical analyses				
Triglycerides (mmol/L)	1.2 (0.7)	1.1 (0.6)	1.2 (0.8)	0.0021
Total cholesterol (mmol/L)	5.6 (1.7)	5.9 (1.3)	5.4 (1.9)	<0.0001
HDL cholesterol (mmol/L)	1.28 (0.54)	1.35 (0.55)	1.25 (0.52)	0.0038
LDL cholesterol (mmol/L)	3.6 (1.6)	3.9 (1.1)	3.4 (1.6)	<0.0001
Fasting plasma glucose (mmol/L)	5.9 (1.2)	5.6 (0.9)	6 (1.3)	<0.0001

a Continuous variables are presented as median (interquartile range). Categorical variables are presented as number (percentage).

b Mann–Whitney *U* test for continuous variables. Chi-square test for categorical variables.

### Clinical and biochemical associations with circulating GDF-15

To examine within-group relationships between GDF-15 and associated clinical and biochemical quantities, quartiles of GDF-15 were generated for both the apparently healthy and unhealthy groups. Among apparently healthy participants, increasing GDF-15 quartile was associated with age (*P* < 0.0001), systolic blood pressure (*P* = 0.0059), NT-proBNP (*P* < 0.0001), and cystatin C (*P* < 0.0001). No significant associations were found between increasing GDF-15 quartile and BMI, sex, smoking status, diastolic blood pressure, or C-reactive protein (CRP) (Table 2; Supplementary Figure 2).

**Table 2.** Clinical and biochemical factors by quartiles of GDF-15 in apparently healthy participants.

		GDF-15 quartile (ng/L)				
		Q1	Q2	Q3	Q4	
Variable^a^	Total	(400–654)	(656–897)	(897–1154)	(1159–3950)	*P* value^b^
Number	268	67	67	67	67	
Demographics						
Age (years)	63.5 (14)	55 (10.5)	63 (12)	67 (10)	70 (12)	<0.0001
Body mass index (kg/m^2^)	25.2 (4)	25.6 (3.7)	24.8 (3.7)	25.4 (3.4)	25 (5.1)	0.6698
Sex, female	75 (28)	13 (19.4)	20 (29.9)	23 (34.3)	19 (28.4)	0.2717
Medical history						
Current or previous smoker	138 (51.5)	35 (52.2)	31 (46.3)	36 (53.7)	36 (53.7)	0.7974
Systolic blood pressure (mmHg)	140 (24.3)	131 (21.3)	141.5 (20.5)	143 (23)	144 (31)	0.0059
Diastolic blood pressure (mmHg)	80 (11)	80 (11.3)	80 (11)	79 (9)	80 (10)	0.6224
Biochemical analyses						
NT-proBNP (ng/L)	57 (68.25)	44 (45)	51 (54)	57 (69.5)	89 (126)	<0.0001
CRP (mg/L)	1.2 (1.51)	1.1 (1.13)	1.1 (1.25)	1.2 (1.25)	1.5 (2.02)	0.1011
Cystatin C (mg/L)	1.04 (0.19)	1 (0.15)	0.98 (0.2)	1.06 (0.15)	1.15 (0.23)	<0.0001

a Continuous variables are presented as median (interquartile range). Categorical variables are presented as number (percentage).

b Kruskal–Wallis test for continuous variables. Chi-square test for categorical variables.

CRP = C-reactive protein; NT-proBNP = N-terminal pro-brain natriuretic peptide.

Among unhealthy participants, increasing GDF-15 quartile was associated with age (*P* < 0.0001), diastolic blood pressure (*P* = 0.0012), NT-proBNP (*P* < 0.0001), CRP (*P* = 0.0025), and cystatin C (*P* < 0.0001). Furthermore, increasing GDF-15 quartile was associated with the majority of clinical exclusion criteria used to select the apparently healthy participants. No significant associations were observed between increasing GDF-15 quartile and BMI, sex, smoking status, or systolic blood pressure (Table 3; Supplementary Figure 3).

**Table 3.** Clinical and biochemical factors by quartiles of GDF-15 in unhealthy participants.

		GDF-15 quartile (ng/L)				
		Q1	Q2	Q3	Q4	
Variable^a^	Total	(417–854)	(855–1186)	(1187–1627)	(1632–11063)	*P* value^b^
Number	542	136	135	136	135	
Demographics						
Age (years)	70 (13)	61.5 (12)	69 (13)	72 (10)	75 (8)	<0.0001
BMI (kg/m^2^)	26.9 (5)	26.9 (4.6)	26.7 (5)	26.8 (4.8)	27.1 (5.6)	0.8573
Sex, female	163 (30.1)	44 (32.4)	49 (36.3)	36 (26.5)	34 (25.2)	0.1580
Medical history						
Current or previous smoker	324 (59.8)	74 (54.4)	79 (58.5)	90 (66.2)	81 (60)	0.2576
Systolic blood pressure (mmHg)	147.8 (26.5)	144.5 (25.9)	148 (26)	149 (28.3)	149 (30)	0.2184
Diastolic blood pressure (mmHg)	80 (16)	84 (14)	80 (12.5)	80 (17.3)	79.5 (14.8)	0.0012
Hypertension	375 (69.2)	90 (66.2)	94 (69.6)	100 (73.5)	91 (67.4)	0.5736
Diabetes mellitus	75 (13.8)	4 (2.9)	9 (6.7)	17 (12.5)	45 (33.3)	<0.0001
Non-sinus rhythm	37 (6.8)	4 (2.9)	7 (5.2)	9 (6.6)	17 (12.6)	0.0125
Major ECG changes	134 (25.1)	28 (20.9)	25 (19.2)	38 (27.9)	43 (32.3)	0.0478
Angina pectoris	56 (10.3)	8 (5.9)	12 (8.9)	14 (10.3)	22 (16.3)	0.0385
Myocardial Infarction	32 (5.9)	0 (0)	10 (7.4)	12 (8.8)	10 (7.4)	0.0084
Heart failure	21 (3.9)	0 (0)	2 (1.5)	8 (5.9)	11 (8.1)	0.0013
LVEF <45%	20 (3.7)	1 (0.7)	4 (3)	7 (5.2)	8 (6)	0.0967
Significant valvular disease	11 (2)	2 (1.5)	4 (3)	3 (2.2)	2 (1.5)	0.7939
TIA/stroke	47 (8.7)	4 (2.9)	11 (8.1)	13 (9.6)	19 (14.1)	0.0129
Peripheral artery disease	70 (12.9)	12 (8.8)	11 (8.1)	17 (12.5)	30 (22.2)	0.0017
Cancer	77 (14.2)	9 (6.6)	18 (13.3)	24 (17.6)	26 (19.4)	0.0130
Biochemical analyses						
NT-proBNP (ng/L)	104.5 (189.5)	62.5 (68)	108 (139.5)	145 (237.8)	167 (428.5)	<0.0001
CRP (mg/L)	1.55 (2.23)	1.25 (1.53)	1.4 (1.75)	1.8 (2.53)	1.8 (2.97)	0.0025
Cystatin C (mg/L)	1.12 (0.24)	1.03 (0.18)	1.09 (0.19)	1.17 (0.23)	1.23 (0.34)	<0.0001

a Continuous variables are presented as median (interquartile range). Categorical variables are presented as number (percentage).

b Kruskal–Wallis test for continuous variables. Chi-square test for categorical variables.

CRP: C-reactive protein; ECG: electrocardiographic; LVEF: left ventricular ejection fraction; NT-proBNP: N-terminal pro-brain natriuretic peptide; TIA: transient ischemic attack.

### Age-dependent reference values for GDF-15 in apparently healthy older adults

**Figure 1.**
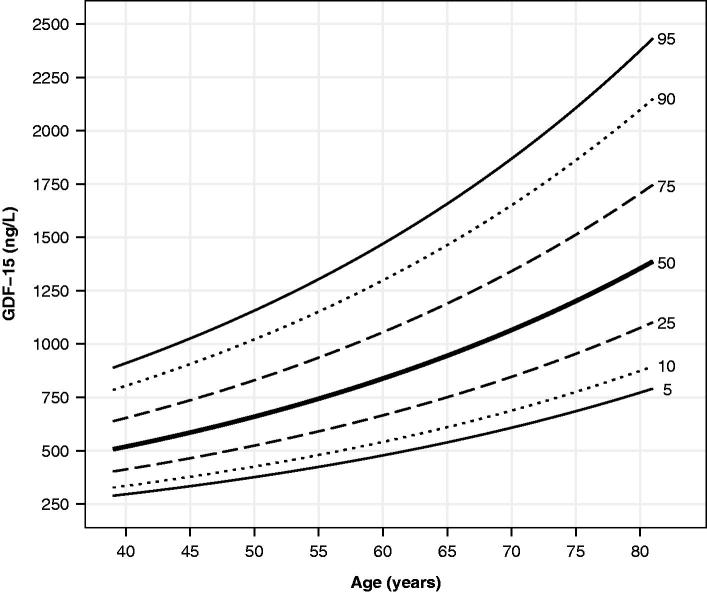
Smoothed centile curves showing circulating GDF-15 concentration by age in apparently healthy participants (*n* = 286).

Reference values of GDF-15 by age for healthy participants are displayed as centile curves in Figure 1 (corresponding tabulated values are presented in Supplementary Table 1). The range of expected values broadened with age and centile magnitude. Expected values at the 50th percentile differed more than two-fold between the oldest and youngest healthy participants. Moreover, there was an approximately three-fold difference between the 95th and 5th centiles observed across ages. Ten-fold cross-validation of the GAMLSS model that generated the age-dependent GDF-15 centiles showed a high degree of similarity in model performance. Namely, the −2 × log(likeli-hood) value of the model trained on all 268 observations was 3832.7, and the cross-validated −2 × log(likelihood) was 3837.0.

### Prognostic impact of GDF-15 reference values

**Figure 2.**
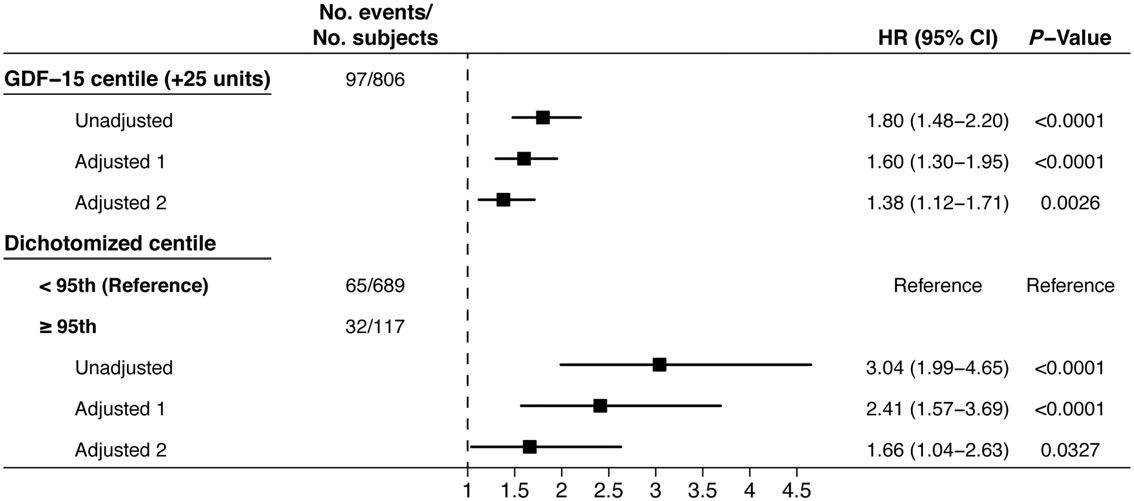
Uni- and multivariable Cox regression models by continuous and dichotomized age-dependent GDF-15 centile and all-cause mortality. Adjusted model 1 is adjusted for age and sex. Adjusted model 2 is adjusted for age, sex, smoking status, logNT-proBNP, and cystatin C.

A total of 97 all-cause deaths were observed during follow-up in 806 participants with complete values for adjusted survival analysis. To evaluate the prognostic impact of GDF-15 reference values established from a population of apparently healthy older adults, age-dependent GDF-15 centile values were used to predict all-cause mortality (Figure 2). In unadjusted Cox regression analysis, GDF-15 centile values were strongly associated with risk of death (per 25 unit increase; HR, 1.80; 95% CI, 1.48–2.20; *P* < 0.0001). GDF-15 centile values remained strongly predictive of all-cause mortality when adjusted for age and sex (per 25 unit increase; HR, 1.60; 95% CI, 1.30–1.95; *P* < 0.0001) and further adjusted for age, sex, smoking status, logNT-proBNP, and cystatin C (per 25 unit increase; HR, 1.38; 95% CI, 1.12–1.70; *P* = 0.0026).

**Figure 3.**
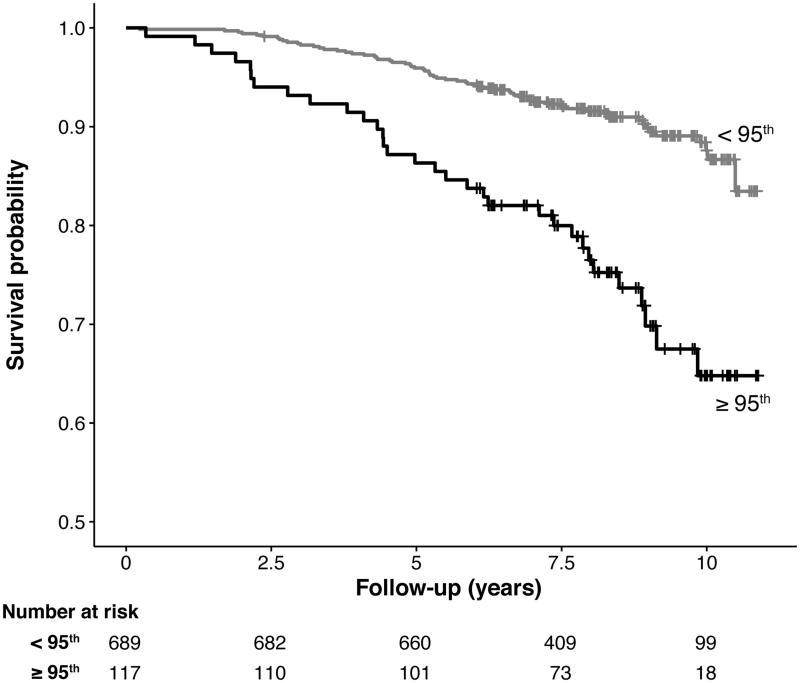
Kaplan–Meier survival curve for all study participants stratified by GDF-15 centile value dichotomized at the 95th centile. Log-rank chi-square = 29.4, *P* < 0.0001.

Additionally, GDF-15 centile values were dichotomized at the 95th centile to model an upper limit for the reference values. Kaplan–Meier analysis yielded a highly significant difference in the survival curves between the dichotomized groups (log-rank chi-square = 29.4, *P* < 0.0001) (Figure 3). Subsequent Cox regression analysis yielded a consistently strong association between GDF-15 centile values greater than or equal to 95 and all-cause mortality. Individuals at or above the 95th centile had a significantly higher risk of death in unadjusted analysis (HR, 3.04; 95% CI, 1.99–4.65; *P* < 0.0001), and this association remained significant when adjusted for age and sex (HR, 2.41; 95% CI, 1.57–3.69; *P* < 0.0001) and adjusted for age, sex, smoking status, logNT-proBNP, and cystatin C (HR, 1.66; 95% CI, 1.04–2.63; *P* = 0.0327) (Figure 2).

## Discussion

In the present study, we determined age-dependent reference values for circulating GDF-15 in a population of apparently healthy older adults. Moreover, we found that GDF-15 reference values were strongly predictive of all-cause mortality when treated as continuous or categorical measures. GDF-15 centiles remained an independent predictor of all-cause mortality when adjusted for age, sex, smoking status, logNT-proBNP, and cystatin C. To our knowledge, this is the first study to provide continuous reference values for GDF-15 in apparently healthy older adults and demonstrate a robust association between reference values and risk of all-cause mortality.

Circulating GDF-15 is well-known to increase with age. Therefore, it is important to develop age-dependence reference limits to realize the clinical implementation of GDF-15 measurements. Such reference limits should be developed from a community-dwelling, and ideally healthy, population. Previous studies have reported levels of circulating GDF-15 in community-dwelling (16,19,20) and apparently healthy (2) populations. However, to our knowledge, no in-depth characterization of the GDF-15 distribution in these studies has been conducted beyond conventional summary statistics, and no age-dependent summaries have been reported. A 2007 study by Kempf and colleagues (2) proposed a GDF-15 concentration of 1200 ng/L as the upper reference limit for elderly individuals. Despite the widespread use of this cutoff value in subsequent studies, we submit that this approach obscures potentially valuable age-dependent fluctuations in GDF-15. Alternatively, we provide age-dependent reference values derived from an evidently healthy population to establish a framework for risk-stratification studies.

In both the apparently healthy and unhealthy groups of our community-based study population, we found associations between GDF-15 quartile and age, blood pressure, NT-proBNP, and cystatin C. Additionally, GDF-15 was associated with a variety of chronic diseases and cardiovascular pathologies among the unhealthy participants. These findings are in agreement with consistent reports of association between GDF-15 and numerous risk factors and biomarkers for CVD (3). From a mechanistic perspective, these broad associations fail to shed light on the precise physiology of GDF-15 but are perhaps informative nonetheless. It has been speculated that GDF-15 is best characterized as a generalized marker for oxidative stress, aging, and/or cellular damage (3,9).

This classification is supported by consistent findings that GDF-15 is associated with both disease-specific outcomes and all-cause mortality in the setting of various conditions, including cancer (21,22), stable coronary heart disease (14), atrial fibrillation (23,24), and acute coronary syndrome (10,25). While the majority of investigation between GDF-15 and all-cause mortality has indeed been conducted in the setting of existing CVD, the community-based Rancho Bernardo (16) and Framingham Heart (19) studies found associations between GDF-15 and all-cause mortality comparable to the present study. To that end, our findings corroborate previous work identifying GDF-15 as an independent risk factor for all-cause mortality.

### Strengths

There are several strengths of the present study. First, we selected a group of apparently healthy older adults without major disease history, vascular and cardiac pathologies, or regular medication use for cardiovascular disease. Given these selection criteria, the subsequent GDF-15 reference values derived from this group reflect a relatively high standard of health against which other populations can be compared. The concentrations of circulating GDF-15 detected in our study are similar to other community-based studies (2,16,19,26). However, GDF-15 is often reported to not be normally distributed, thereby limiting comparisons across studies using conventional summary statistics. Therefore, we utilized a GAMLSS modeling approach, which has been used by the World Health Organization to develop its child growth standards (27), to provide a more precise characterization of the GDF-15 distribution in our population by establishing age-dependent centile values. Additional strengths of our study include a relatively long follow-up time and one centralized location for plasma storage and analysis of GDF-15.

### Limitations

Several limitations of this study should be mentioned. First, several important diagnoses (hypertension, heart failure, cancer) used to select the ‘apparently healthy’ group were only available as self-reported data, thereby introducing the possibility of recall bias in group selection. Additionally, the relatively small number of all-cause deaths observed during follow-up potentially reduces the power of our survival analysis. Lastly, the study population is composed entirely of Caucasian older adults, thereby limiting the generalizability of our results to other populations of more diverse age or ethnic composition.

## Conclusions

In conclusion, age-dependent reference values for circulating GDF-15 were developed from a population of apparently healthy older adults. GDF-15 levels expressed as age-dependent centiles were strongly associated with all-cause mortality in a community-based sample of older adults. Therefore, GDF-15 reference values could be a useful tool for risk-stratification in a clinical setting.
